# Supplementary material for: Instability in NAD+ metabolism leads to impaired cardiac mitochondrial function and communication
Source: eLife. 2021 Aug 3;10:e59828. doi: 10.7554/eLife.59828 (PMC8331182; doi:10.7554/eLife.59828)
Supplement: Source data 4. [file elife-59828-data4.pdf]

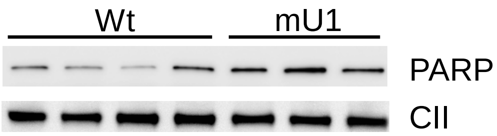

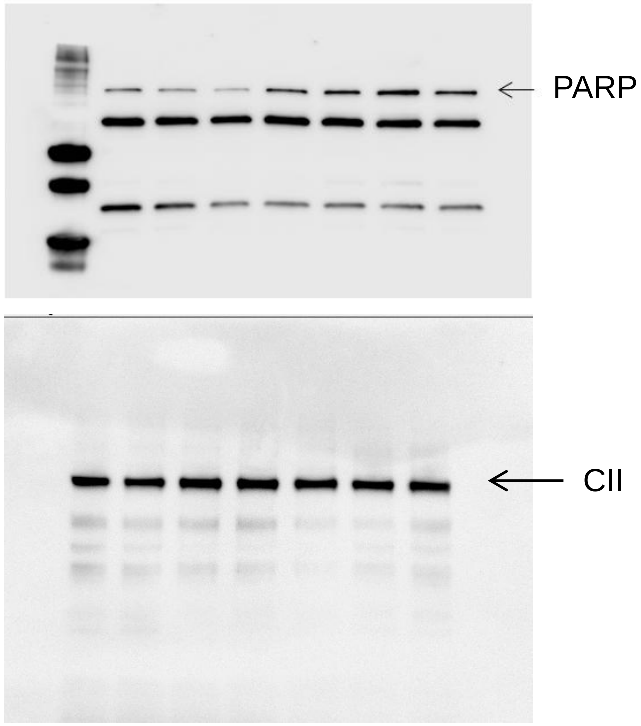

Fig 1A

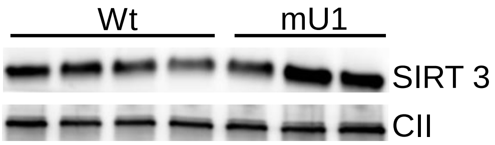

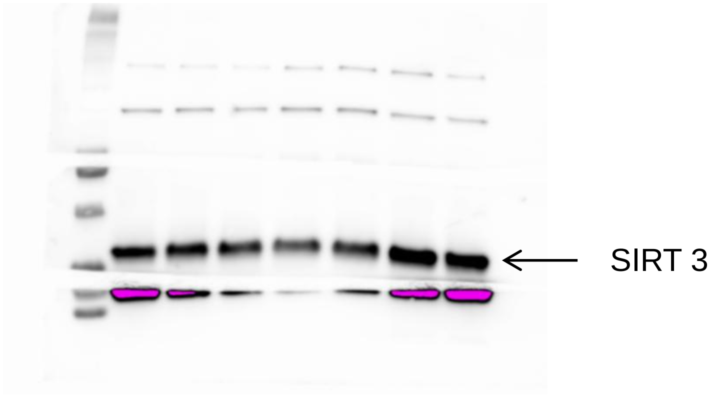

(Blot was cut in two)

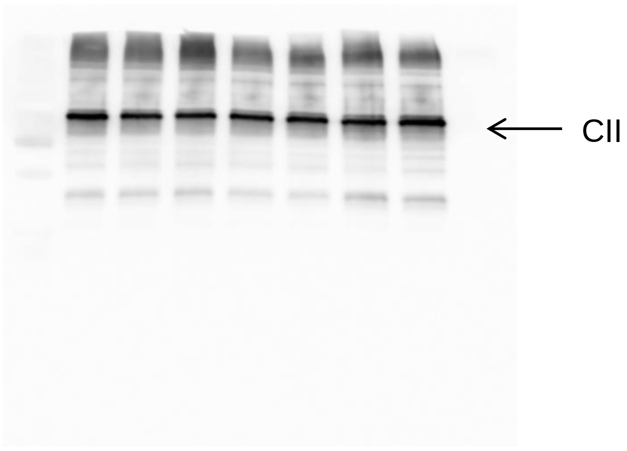

Fig 1D

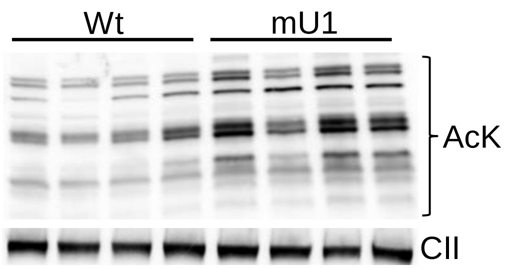

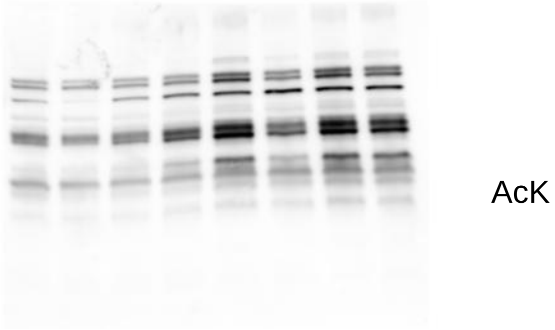

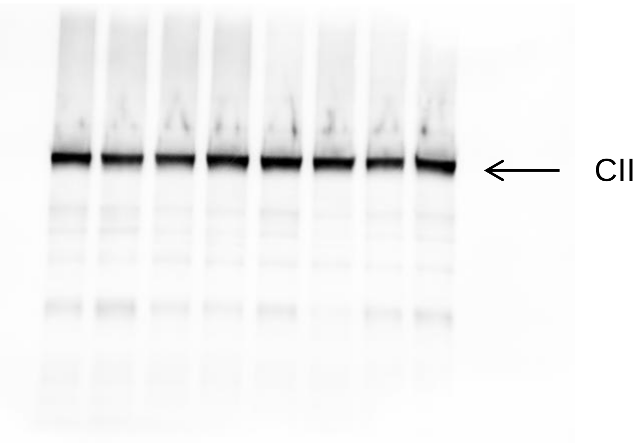

Fig 1F

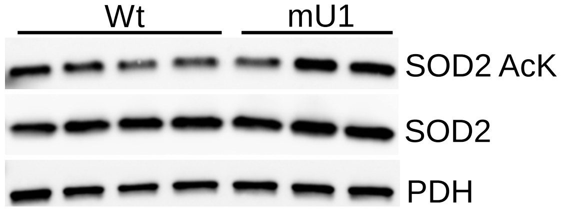

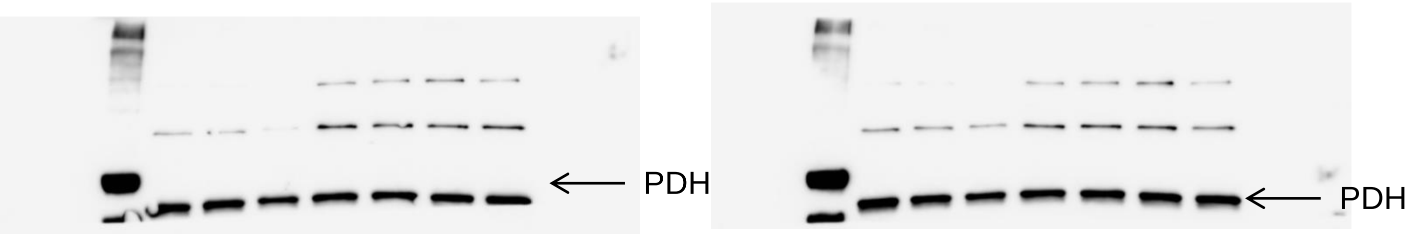

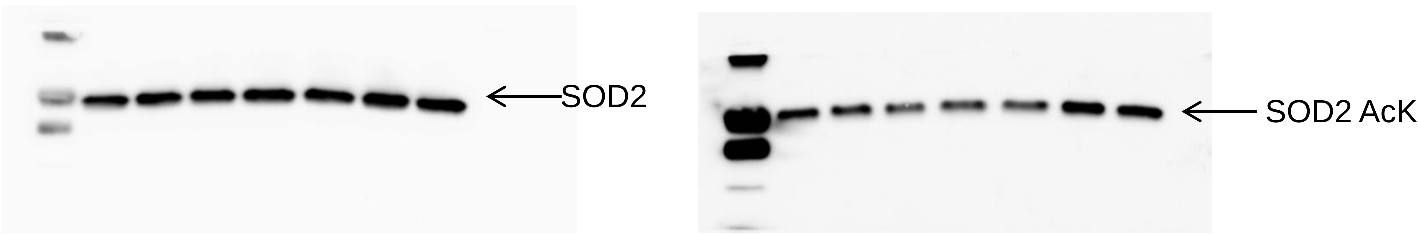

(Blot(s) was cut in two)

Fig 1H

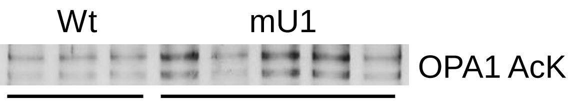

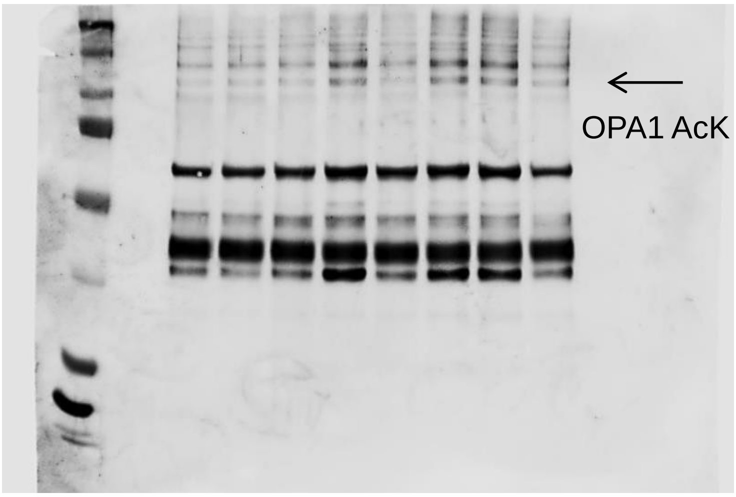

Fig 1J

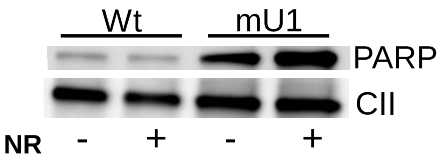

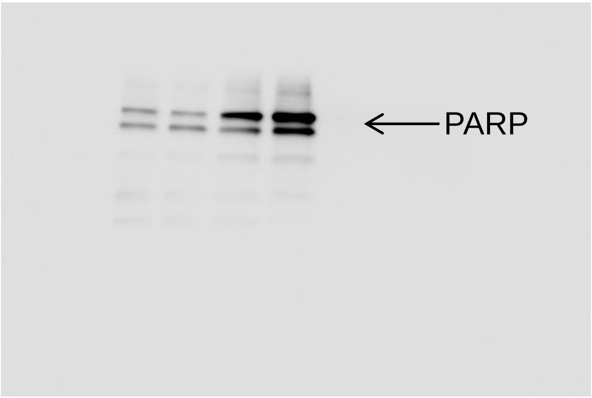

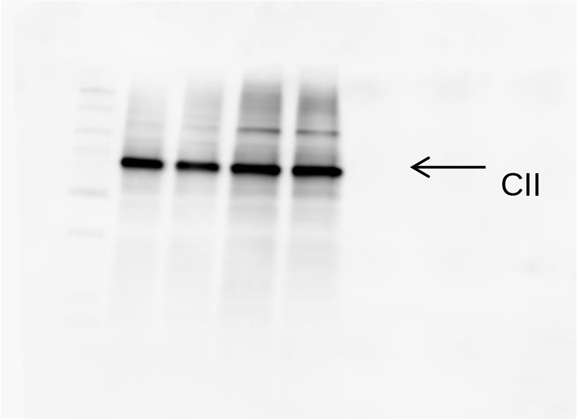

Fig 6A

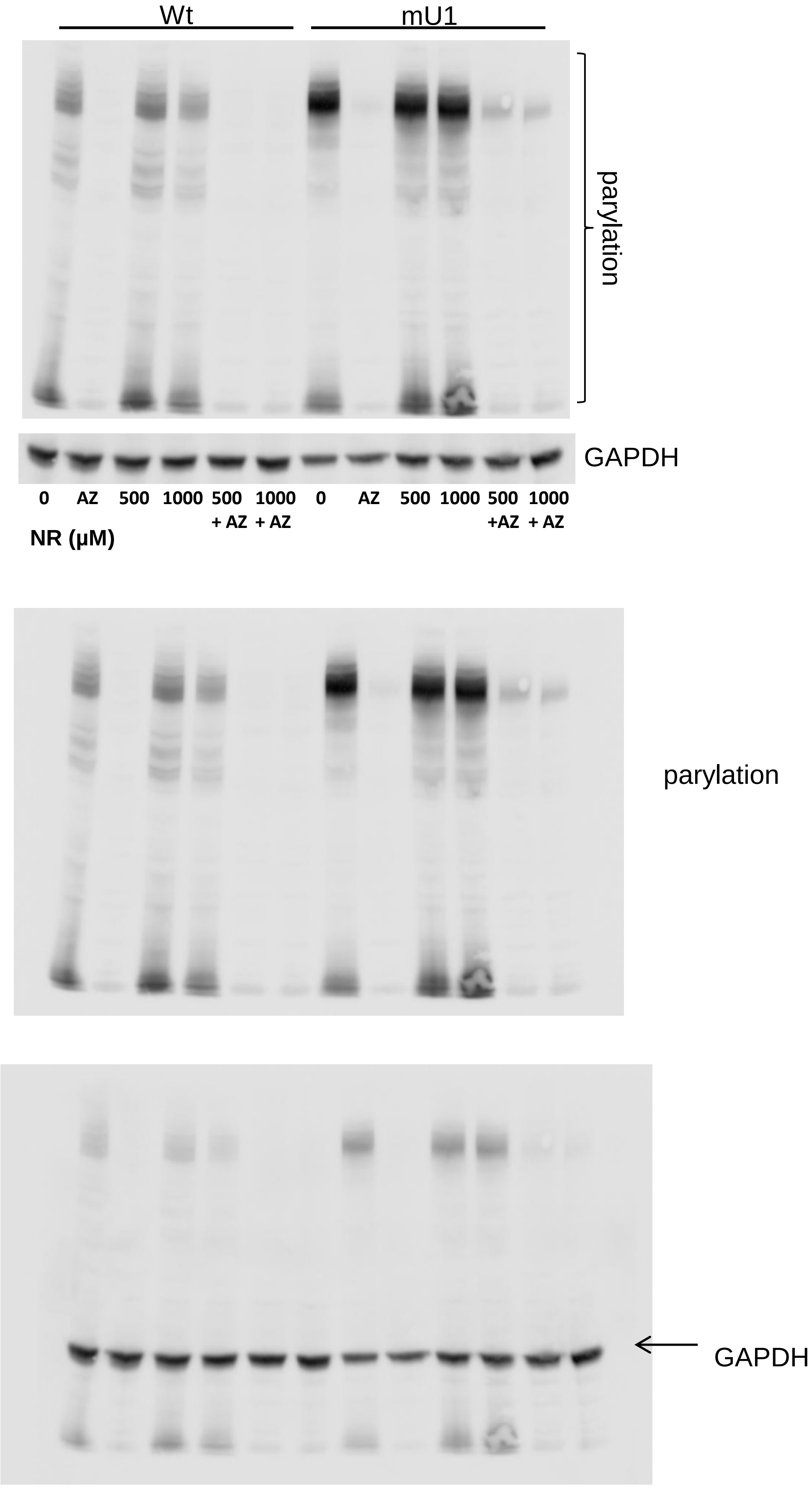
Wt
mU1
parylation
GAPDH
0 AZ 500 1000 500 + AZ 1000 + AZ 0 AZ 500 1000 500 +AZ 1000 + AZ
NR (μM)
parylation
GAPDH

Fig 6C

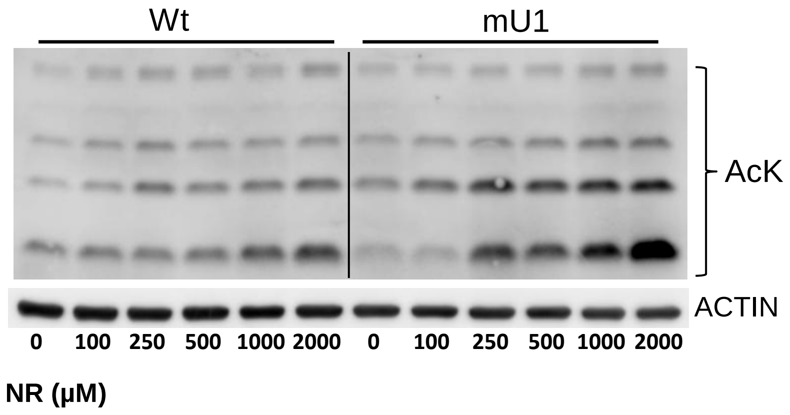

Wt
mU1
AcK
ACTIN
0 100 250 500 1000 2000 0 100 250 500 1000 2000
NR (µM)

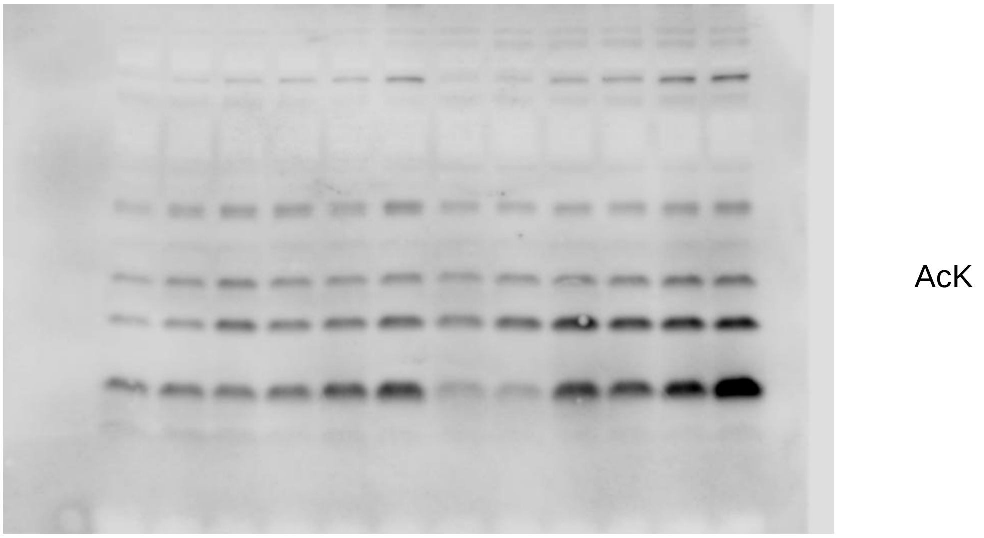

AcK

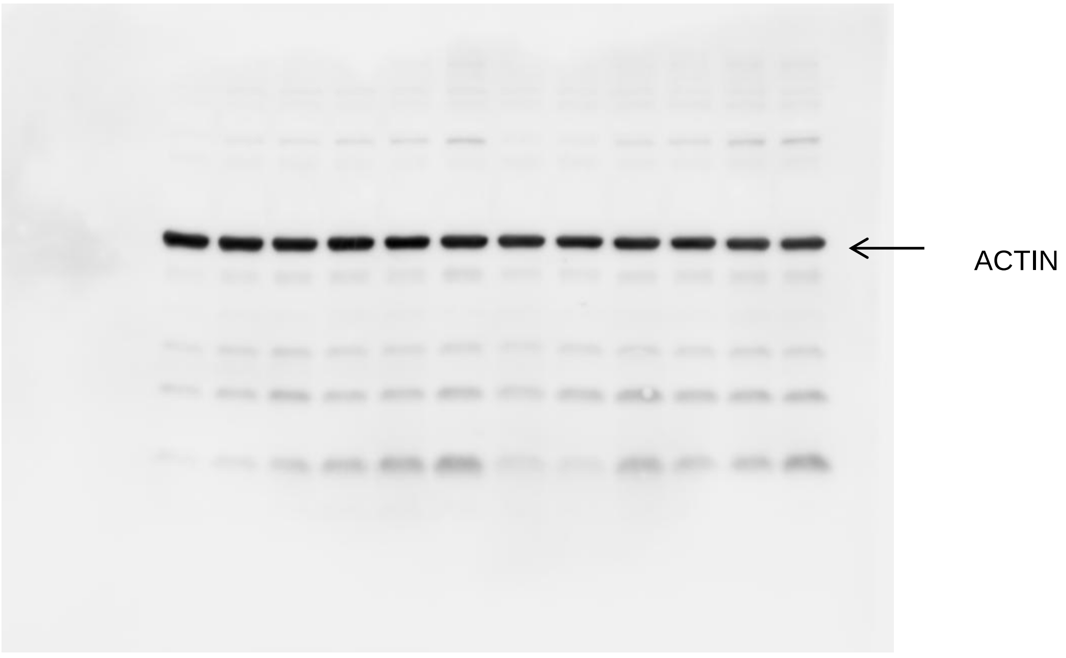

ACTIN

Fig 6E

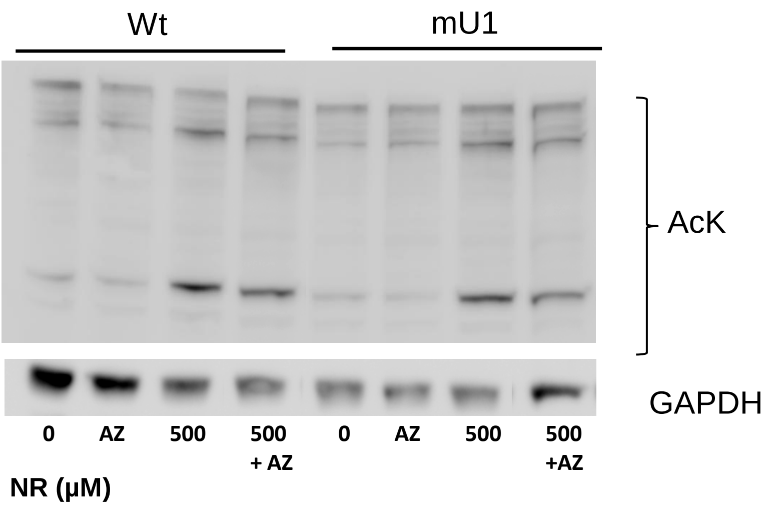

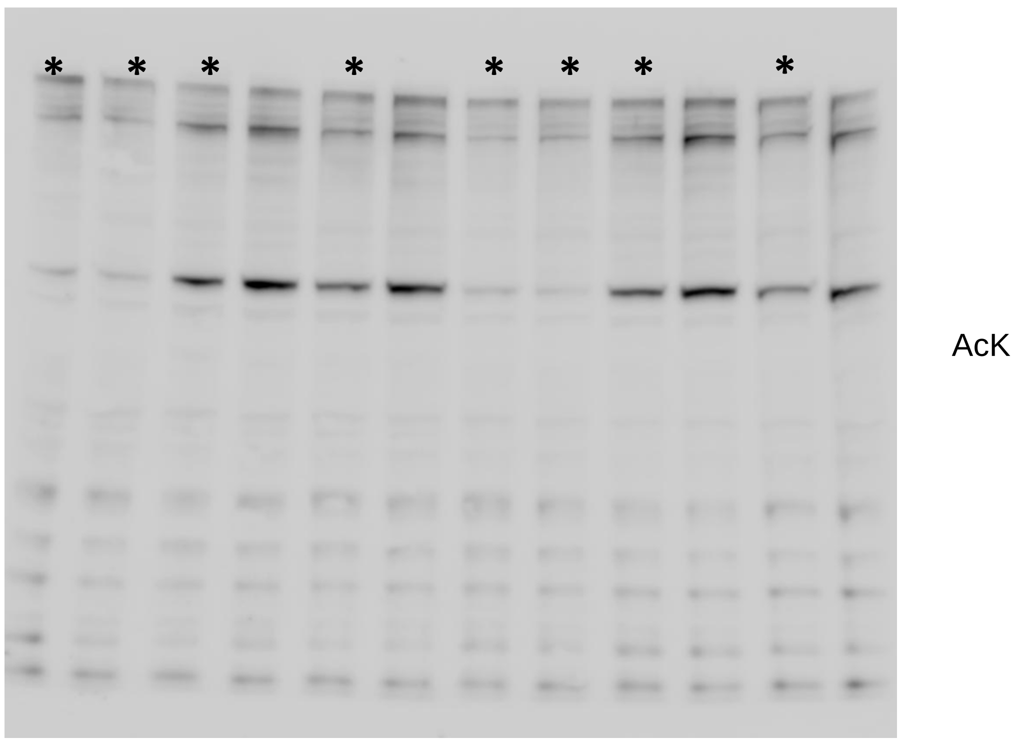

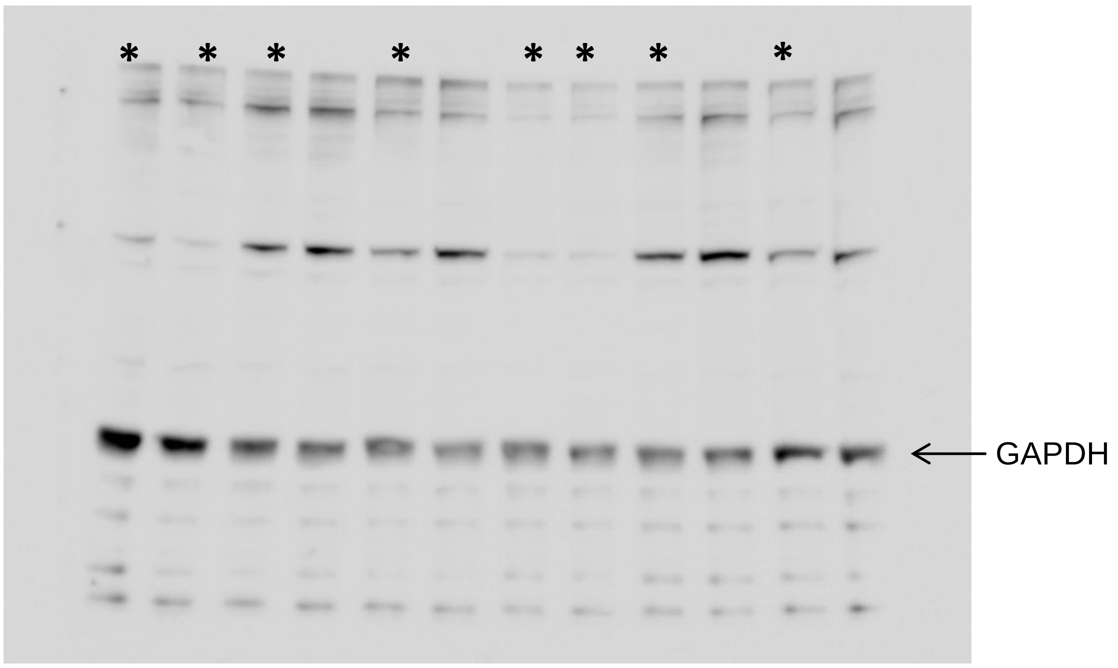

Fig 6G

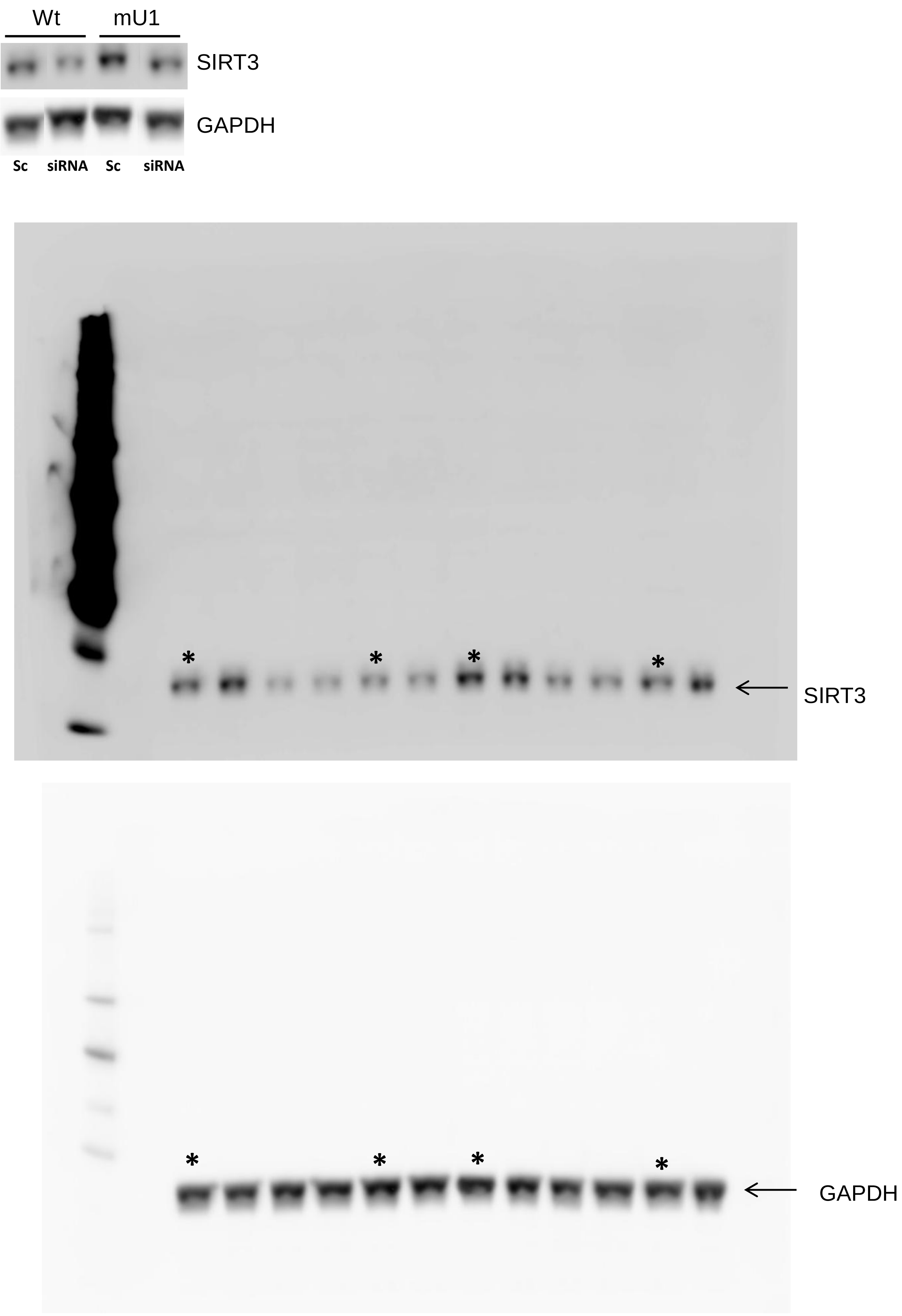

Fig 6I

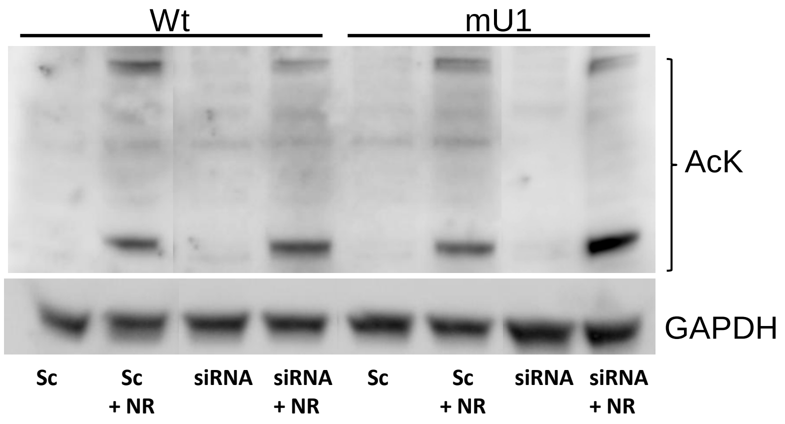
Wt
mU1
AcK
GAPDH
Sc
Sc + NR
siRNA
siRNA + NR
Sc
Sc + NR
siRNA
siRNA + NR

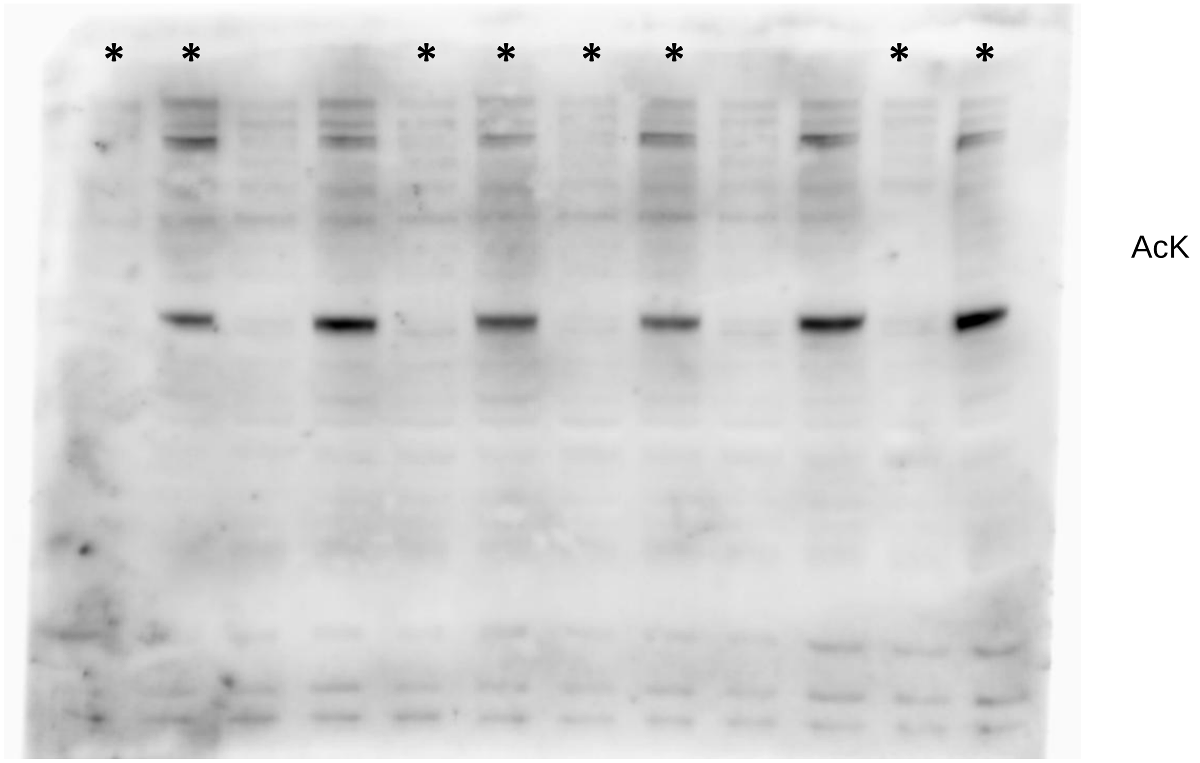
AcK

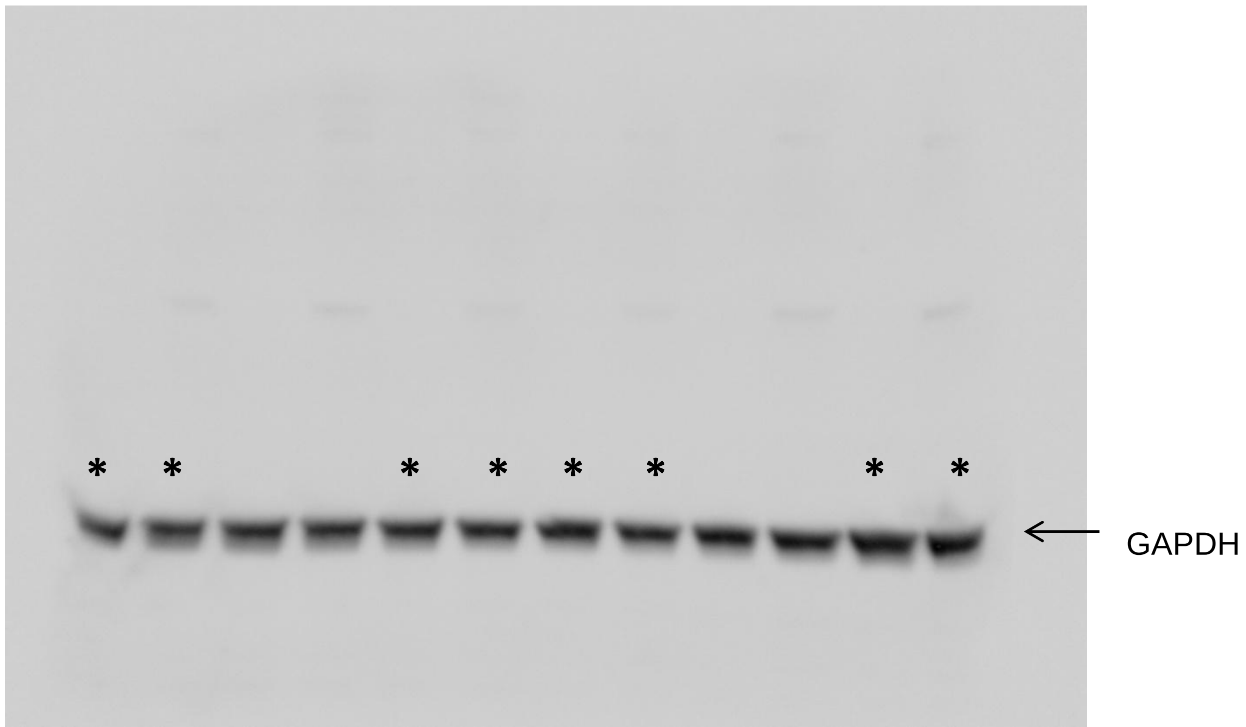
GAPDH
