# Supplementary material for: Instability in NAD+ metabolism leads to impaired cardiac mitochondrial function and communication
Source: eLife. 2021 Aug 3;10:e59828. doi: 10.7554/eLife.59828 (PMC8331182; doi:10.7554/eLife.59828)
Supplement: Supplementary file 1. [file elife-59828-supp1.docx]

| **Protein** | **Gene Symbol** | **Cellular Component (GO)** |
| --- | --- | --- |
| Q8K1C0 | Angel2 | GO:0015030 Cajal body;GO:0016604 nuclear body;GO:0044451 nucleoplasm part |
| Q99L43 | Cds2 | GO:0005789 endoplasmic reticulum membrane;GO:0098827 endoplasmic reticulum subcompartment;GO:0042175 nuclear outer membrane-endoplasmic reticulum membrane network |
| A3KFM7 | Chd6 | GO:0005654 nucleoplasm;GO:0031981 nuclear lumen;GO:0070013 intracellular organelle lumen |
| P63242 | Eif5a | GO:0005642 annulate lamellae;GO:0005643 nuclear pore;GO:0098589 membrane region |
| Q8BGY2 | Eif5a2 | GO:0005643 nuclear pore;GO:0005635 nuclear envelope;GO:0031967 organelle envelope |
| P04117 | Fabp4 | GO:0005654 nucleoplasm;GO:0005829 cytosol;GO:0031981 nuclear lumen |
| Q9D1G3 | Hhatl | GO:0048471 perinuclear region of cytoplasm;GO:0005783 endoplasmic reticulum;GO:0012505 endomembrane system |
| Q9Z2G9 | Htatip2 | GO:0005635 nuclear envelope;GO:0031967 organelle envelope;GO:0031975 envelope |
| Q07113 | Igf2r | GO:0005641 nuclear envelope lumen;GO:0030140 trans-Golgi network transport vesicle;GO:0030118 clathrin coat |
| Q9CPU4 | Mgst3 | GO:0005635 nuclear envelope;GO:0031967 organelle envelope;GO:0031975 envelope |
| Q11011 | Npepps | GO:0005829 cytosol;GO:0005634 nucleus;GO:0044444 cytoplasmic part |
| Q5M8N4 | Sdr39u1 | (Human)GO:0005634 nucleus;GO:0043231 intracellular membrane-bounded organelle;GO:0043227 membrane-bounded organelle |
| Q9D666 | Sun1 | GO:0034992 microtubule organizing center attachment site;GO:0034993 meiotic nuclear membrane microtubule tethering complex;GO:0106083 nuclear membrane protein complex |
| Q8BJS4 | Sun2 | GO:0034992 microtubule organizing center attachment site;GO:0034993 meiotic nuclear membrane microtubule tethering complex;GO:0106083 nuclear membrane protein complex |
| Q3TMP8 | Tmem38a | GO:0016529 sarcoplasmic reticulum;GO:0016528 sarcoplasm;GO:0031965 nuclear membrane |
| Q8C0L0 | Tmx4 | GO:0005637 nuclear inner membrane;GO:0031965 nuclear membrane;GO:0005635 nuclear envelope |
| P99024 | Tubb5 | GO:0045298 tubulin complex;GO:0005641 nuclear envelope lumen;GO:0031970 organelle envelope lumen |

Supplementary file 1: Table of GO terms from proteomic analysis
